# An Introduction to Practical Pharmacy; with Many Formulæ and Prescriptions

**Published:** 1860-01

**Authors:** 


ART. IV.—*An Introduction to Practical Pharmacy; designed as a Text-book for the Student, and as a Guide to the Physician and Pharmaceutist. With many Formulæ and Prescriptions.* By EDWARD PARRISH, Graduate in Pharmacy, Member of the Philadelphia College of Pharmacy, and of the American Pharmaceutical Association, and Principal of the School of Practical Pharmacy, Philadelphia. Second edition. With 246 Illustrations. 8vo., pp. 720. Blanchard & Lea: Philadelphia, 1859.

THE medical and pharmaceutical professions of the United States are indebted to Mr. Parrish for the first systematic treatise on practical pharmacy ever published on this side of the Atlantic. That the work has been well executed is sufficiently attested by the demand of a second edition so soon after the appearance of the first. The present issue has been enriched by nearly two hundred pages of new matter, and by a number of original engravings, rendered necessary by the rapid progress of pharmacy within the past few years. To impart to it as much completeness as possible, the author has availed himself of many recent foreign works, not generally accessible to students; and he has spared no pains to make it a fair and satisfactory exponent of the existing state of the science. The whole treatise is eminently practical; and there is no production of the kind in the English language so well adapted to the wants of the pharmaceutist and druggist. To physicians, also, it cannot fail to be highly valuable, especially to those who are obliged to prepare and compound many of their own medicines; a practice which, in view of the numerous mistakes committed by uneducated and unscrupulous apothecaries, is at the present day unfortunately too much neglected.

The work of Mr. Parrish is well written; and we cannot doubt that the present edition will, like its predecessor, have an extensive circulation. The eminent publishers deserve great credit for the manner in which it has been issued. They have evidently spared neither pains nor money to do justice to their author's labors.

